# The impact of zinc supplementation on carbapenem MICs among bacteria expressing IMP metallo-beta-lactamase

**DOI:** 10.1099/acmi.0.000972.v4

**Published:** 2025-06-26

**Authors:** Susan V. Grooters, Dixie F. Mollenkopf, Gregory A. Ballash, Thomas E. Wittum

**Affiliations:** 1 Department of Veterinary Preventive Medicine, The Ohio State University, Columbus, Ohio, USA

**Keywords:** antimicrobial resistance, antimicrobial susceptibility testing, *bla*_IMP_, carbapenemase, metallo-beta-lactamase, zinc

## Abstract

Antibiotic-resistant infections cause an estimated 2.8 million illnesses and 35,900 deaths annually in the USA. Carbapenems are a class of antibiotics that are generally reserved to treat life-threatening invasive infections including sepsis. Accurate diagnosis of carbapenem-resistant infections is critical for early and appropriate treatment. *bla*_IMP_ encodes bacterial production of the IMP metallo-beta-lactamase (MBL), which can confer resistance to all the beta-lactams including carbapenems. Zinc is an essential co-factor in the IMP MBL enzymatic hydrolysis of carbapenems. Tests for the presence of IMP carbapenemase, such as the Carba NP, include zinc sulphate (ZnSO_4_) although broth dilution methods for determining MIC for carbapenems may vary. We hypothesized that ZnSO_4_ availability would improve the accuracy of carbapenem MIC determination for bacteria expressing *bla*_IMP_. Thus, the objective of this study was to determine if supplemental ZnSO_4_ affects the carbapenem MICs of *Enterobacterales*, *Alteromonadales* and *Moraxellales* expressing *bla*_IMP_. Isolates utilized for this study were originally recovered from environmental samples collected at farms, wastewater treatment plants and surface water. They were selected based on phenotypic non-susceptibility to carbapenems and genetic confirmation of bacterial carriage of *bla*_IMP_. Cation-adjusted Mueller–Hinton broth suspensions of each isolate standardized to a 0.5 MacFarland standard were tested with and without ZnSO_4_ added at 0.1 mmol l^−1^ concentration to determine MICs using standard extended-spectrum beta-lactamase microbroth dilution MIC panels. Although we observed that *Morganellaceae* imipenem MICs were higher (*P*<0.001) than those from other bacteria harbouring *bla*_IMP_, the inclusion of supplemental ZnSO_4_ did not influence carbapenem MIC. This suggests that supplemental ZnSO_4_ will not improve the accuracy of carbapenem MICs in environmental bacteria expressing IMP carbapenemase. Additional research will be required to identify important factors that may influence the expression of carbapenemase including IMP and the accurate determination of clinical MICs, which is critical to appropriate therapeutic decision-making.

**Impact Statement**

Antibiotic-resistant infections have been designated a public health threat by the CDC and a public health crisis by the United Nations’ World Health Organization. Carbapenems are a class of antibiotics often used to treat the deadliest of bacterial infections. The ability to accurately detect bacteria that are resistant to carbapenems is important to both research and clinical decision-making. We hypothesized that accurate carbapenem MIC determination in bacteria expressing the carbapenemase gene for *bla*_IMP_ may require additional available zinc in standard testing media. Our results indicate that the addition of supplemental zinc, in the form of ZnSO_4_, does not affect the phenotypic expression of carbapenem susceptibility among different bacterial taxa from environmental sources that carry *bla*_IMP_ in standard microbroth dilution tests. As a result, supplemental zinc will not be expected to improve the accuracy of MIC determination in environmental bacteria harbouring *bla*_IMP_.

## Data Summary

Sequence reads have been deposited in the NCBI Sequence Read Archive and are available under BioProject accession PRJNA1197433. Individual sequence accession numbers include SAMN45808700, SAMN45808699, SAMN45808692, SAMN45808698, SAMN45808672, SAMN45808671, SAMN45808680, SAMN45808675, SAMN45808685 and SAMN45808687.

## Introduction

In the USA, antibiotic-resistant infections cause an estimated 2.8 million illnesses and 35,900 deaths annually [1]. Of those illnesses, ~9,000 are estimated to be from carbapenem-resistant bacterial infections [1]. The carbapenem antibiotics are generally reserved to treat life-threatening invasive infections including sepsis. Acquired bacterial resistance to carbapenems is often the result of transmissible carbapenemase genes. One such gene, *bla*_IMP_, encodes for the production of the IMP carbapenemase, which can confer reduced susceptibility or resistance to carbapenems and other beta-lactam antibiotics. Bacterial carbapenemase genes are readily transferred horizontally within and between bacterial taxa on mobile genetic elements such as plasmids and transposons [2].

Carbapenemases are classified as serine-beta-lactamases or metallo-beta-lactamases (MBLs) [3]. Class B MBLs can catalyse the hydrolysis of a wide range of beta-lactam antibiotics, including carbapenems [4]. IMP carbapenemases are a subgroup of acquired MBLs of the B1 subclass, with strictly conserved zinc ligand residues and zinc-binding motifs that distinguish them from the other B subclasses [5]. B1 enzymes are most active with two zinc ions bound in the active site and have been shown to have a broad-spectrum substrate profile [5]. *bla*_IMP_ encodes for the production of a B1 MBL, which can confer reduced susceptibility or resistance to carbapenems and other beta-lactam antibiotics. Zinc is an essential co-factor in the MBL enzymatic hydrolysis of the beta-lactam ring of carbapenems, which is why zinc sulphate (ZnSO_4_) is included in the tests for carbapenemase production, including the Carba NP [6].

The impact of differing levels of zinc availability on *in vitro* and *in cell* inhibition of carbapenemase activity by chelation or replacement among clinically relevant MBLs has been reported [7]. Others have indicated that zinc concentration in media is not a driver of allele mutation or increased cell resistance to carbapenems [8]. This suggests that adequate zinc is still essential for carbapenemase activity, particularly for IMP compared with other MBLs, which have adapted to zinc-limited environments [7].

However, an additional source of zinc is not standardized in media utilized for carbapenem MIC determination [9]. If a minimum threshold of available zinc is required for effective hydrolysis of carbapenems by IMP B1 MBLs, then inadequate available zinc in media used for MIC determination of *Enterobacterales* or other bacteria might result in MIC values that are incorrectly low and could inappropriately influence their clinical interpretation. Therefore, the addition of available ZnSO_4_ might improve the accuracy of carbapenem MIC estimation for bacteria producing the IMP MBL.

The impact of zinc concentration in Mueller–Hinton (MH) broth on MICs of MBL-producing *Enterobacteriaceae* has been previously reported [9]. They observed zinc variability among commercial lots of MH broth, resulting in different classifications of meropenem susceptibility among multiple MBL genotypes. They concluded that a consensus on the appropriate amount of zinc in culture media is needed to ensure that variations in the zinc content of commercial media do not significantly influence MBL antimicrobial susceptibility test results [9].

In our laboratory, we have previously observed that different taxonomic families of bacteria, all harbouring the class 2 integron and *bla*_IMP_ gene cassette, can have different carbapenem resistance phenotypes. We have reported that species within the family of *Enterobacteriaceae* isolates expressing *bla*_IMP_ appear fully susceptible to imipenem, while species within the *Morganellaceae* taxonomic family expressing *bla*_IMP_ appear to have reduced susceptibility [10]. This finding was in contrast to MIC data for *Enterobacteriaceae* and *Morganellaceae* producing NDM MBLs, which produce similar carbapenem resistance phenotypes. We hypothesized that these observed differences in carbapenem MICs among bacteria expressing *bla*_IMP_ could be due to a lack of available exogenous zinc. There is relatively limited scientific characterization of bacterial isolates expressing *bla*_IMP_ and the specific alleles that are included in this study, IMP-95, IMP-64 and IMP-27, for which limited MIC values are available [2].

We hypothesized that supplemental ZnSO_4_ in media will improve the accuracy of carbapenem MIC determination for bacteria expressing *bla*_IMP_ and that variability in phenotypic imipenem susceptibility among taxonomic families is attributable to differences in IMP carbapenemase requirements for exogenous zinc availability. Therefore, the objectives of this study are to document the role of exogenous ZnSO_4_ availability on phenotypic imipenem susceptibility of *Morganellaceae*, *Enterobacteriaceae*, *Shewanellaceae* and *Moraxellaceae* isolates producing the IMP MBL.

## Methods

A total of 25 bacterial isolates were utilized in this study that originated from environmental samples: on farms, wastewater treatment plants and surface waters. Selective media used in the initial screening for isolates showed reduced susceptibility of the isolates to meropenem using methods that have been previously described [10,12]. Isolates utilized for this study were all confirmed to produce carbapenemase using the Carba NP test [6]. These isolates represent a relatively rare genotype in the USA and were collected over time beginning in 2016 through 2021, some of which have been previously reported [10,13].

Isolates in this study phenotypically express reduced susceptibility to carbapenems, and bacterial carriage of *bla*_IMP_ was confirmed by PCR and Sanger sequencing of amplicons. Taxonomic family and genus were identified using MALDI-TOF (Bruker Scientific, LLC, Billerica, MA) and sequence-based bioinformatic tools for 17 isolates for which whole-genome sequence data were available [14,15]. The 25 study isolates included 10 *Enterobacteriaceae*, 8 *Morganellaceae*, 5 *Shewanellaceae* and 2 *Moraxellaceae* and were presented by taxonomic family rather than species to be consistent with phenotypic distributions that we previously observed [10]. Sequence data were compared with online databases [16,19] to determine the index number of the *bla*_IMP_ gene allele (Table 1).

**Table 1.** MIC values of 25 bacterial isolates expressing IMP carbapenemase against imipenem (IPM) and meropenem (MEM) with and without supplemental ZnSO_4_

Isolate	Taxonomic family	*bla*_IMP_	MICIPM	MICIPM (*+*Zn)	MICMEM	MICMEM (*+*Zn)
S15-B*	*Morganellaceae*	*bla*_IMP-27_	**4**	**4**	2	**4**
*SF 405*	*Morganellaceae*	*bla*_IMP-64_	**>8**	**8**	**8**	**8**
*S13-19B*	*Morganellaceae*	*bla*_IMP-64_	**8**	**8**	**8**	**8**
*WT2A*	*Morganellaceae*	*bla*_IMP-64_	**>8**	**4**	**>8**	**8**
S5-A*	*Morganellaceae*	*bla*_IMP-27_	**8**	**4**	**4**	**4**
*G1*	*Morganellaceae*	*bla*_IMP-27_	**>8**	2	**>8**	**4**
S4-B*	*Morganellaceae*	*bla*_IMP-27_	**4**	**4**	2	2
*WP 2A*	*Morganellaceae*	*bla*_IMP-27_	2	**4**	**4**	**4**
S8-B*	*Enterobacteriaceae*	*bla*_IMP-27_	≤1	≤1	**8**	**8**
S17*	*Enterobacteriaceae*	*bla*_IMP-27_	≤1	≤1	**4**	**4**
*S15-A*	*Enterobacteriaceae*	*bla*_IMP-64_	≤1	≤1	**4**	**4**
*S13-A*	*Enterobacteriaceae*	*bla*_IMP-64_	≤1	≤1	**8**	**8**
*MU 7A*	*Enterobacteriaceae*	*bla*_IMP-64_	≤1	**8**	**4**	**8**
*S13-19A*	*Enterobacteriaceae*	*bla*_IMP-64_	≤1	≤1	2	2
S23*	*Enterobacteriaceae*	*bla*_IMP-27_	≤1	≤1	**4**	**4**
S13-B*	*Enterobacteriaceae*	*bla*_IMP-27_	≤1	≤1	**4**	**4**
*S11*	*Enterobacteriaceae*	*bla*_IMP-64_	≤1	≤1	≤1	≤1
S18*	*Enterobacteriaceae*	*bla*_IMP-27_	≤1	≤1	2	2
*D5-33A*	*Shewanellaceae*	*bla*_IMP-27_	2	2	8	8
*JP-35A-EFFB*	*Shewanellaceae*	*bla*_IMP-27_	≤1	≤1	4	4
*MD-1B*	*Shewanellaceae*	*bla*_IMP-27_	≤1	≤1	4	4
*MU-14A*	*Shewanellaceae*	*bla*_IMP-27_	≤1	≤1	4	4
*MU-9A*	*Shewanellaceae*	*bla*_IMP-27_	≤1	≤1	4	2
*D4-50A*	*Moraxellaceae*	*bla*_IMP-27_	≤1	≤1	≤1	≤1
*D5-7A*	*Moraxellaceae*	*bla*_IMP-95_	≤1	≤1	**8**	**8**

* Only Sanger sequencing of PCR product confirmation of *bla*_IMP-27_ gene.

Other isolates with allele index numbering are based on whole-genome sequencing. MIC values in bold indicate that an isolate is considered to be clinically resistant to imipenem (IPM) or meropenem (MEM), respectively.

MICs were determined for each isolate using the Sensititre broth microdilution system (Thermo Fisher Scientific, Oakwood Village, OH) following the Clinical and Laboratory Standards Institute (CLSI) guidelines [20,21]. Isolates were processed in duplicate, with and without added ZnSO_4_ at 0.1 mmol l^−1^, the same concentration of zinc present in the Carba NP test [6], in cation-adjusted MH broth (Thermo Fisher Scientific) suspensions to a 0.5 McFarland standard. While we did not measure the zinc concentration of the media and it is not reported by the manufacturer, reports from the literature of zinc concentrations ranging from 0.01 to 0.1 mmol l^−1^ in commercial media suggest that this amount of added ZnSO_4_ will provide significant free zinc available to the cell [22,23]. Standard carbapenem-resistant *Enterobacterales* and extended-spectrum beta-lactamase Sensititre MIC panels CMV3AGNF and GNX2F, respectively (Thermo Fisher Scientific), were utilized to produce paired MIC results for each isolate.

ZnSO_4_ enriched and non-enriched pairs’ MIC data were log_2_ transformed for analysis and compared using the Wilcoxon signed-rank test in Stata IC13 (StataCorp LP, College Station, TX, USA) for paired data. This test evaluated the null hypothesis that the median difference among MIC pairs was zero. The Kruskal–Wallis test in Stata IC13 (StataCorp LP) was used to assess the null hypothesis that there was no difference in MIC between taxonomic groups.

## Results

**Fig. 1.**
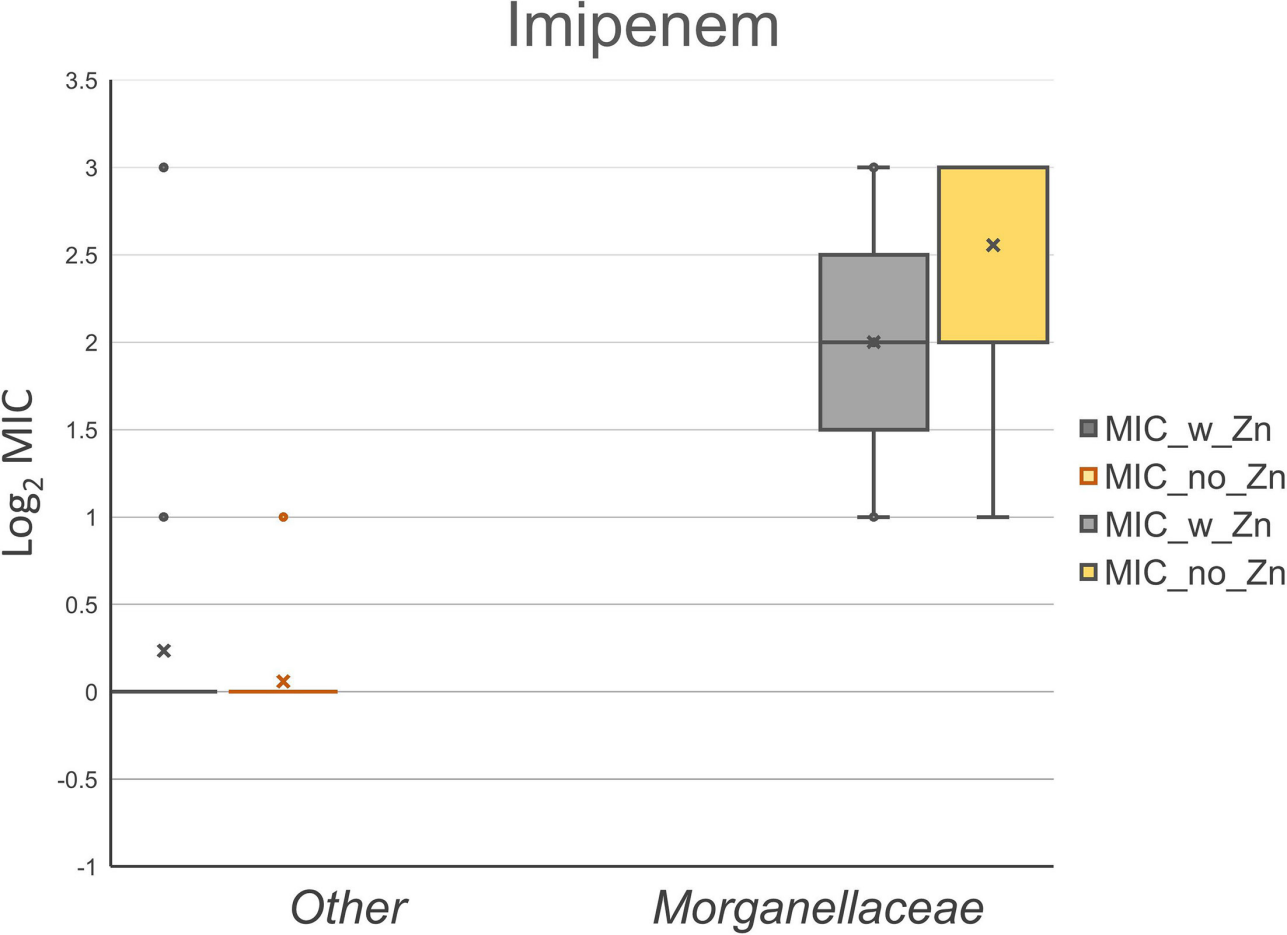
Observed MIC values against imipenem for 8 *Morganellaceae* and 17 other taxonomic family isolates (10 *Enterobacteriaceae*, 5 *Shewanellaceae* and 2 *Moraxellaceae*) expressing IMP carbapenemase with and without supplemental ZnSO_4_. The supplemental ZnSO_4_ did not impact imipenem MIC (*P*>0.05).

**Fig. 2.**
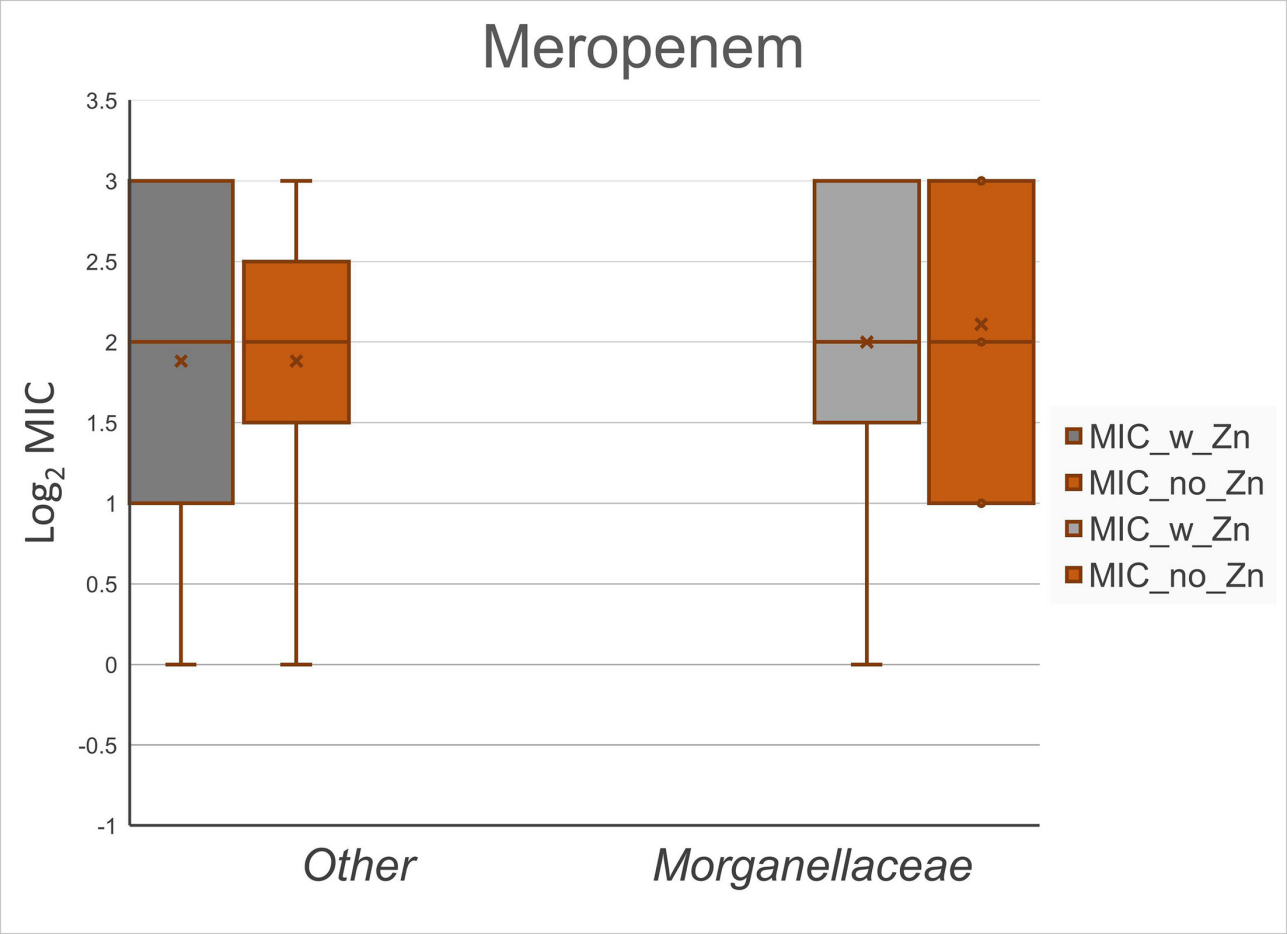
Observed MIC values against meropenem for 8 *Morganellaceae* and 17 other taxonomic family isolates (10 *Enterobacteriaceae*, 5 *Shewanellaceae* and 2 *Moraxellaceae*) expressing IMP carbapenemase with and without supplemental ZnSO_4_. The supplemental ZnSO_4_ did not impact meropenem MIC (*P*>0.05).

**Fig. 3.**
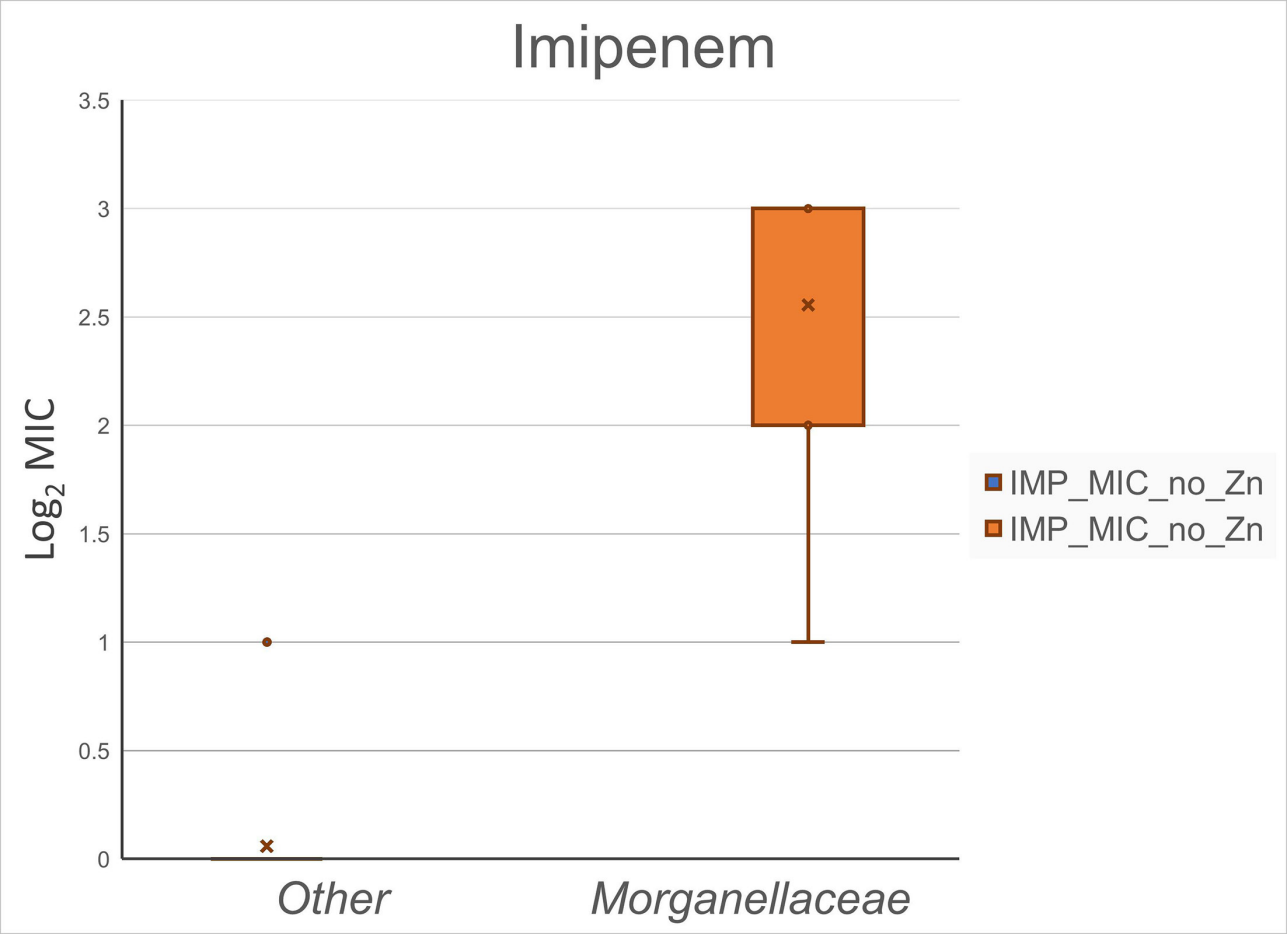
Observed MIC values against imipenem for 8 *Morganellaceae* and 17 other taxonomic family isolates (10 *Enterobacteriaceae*, 5 *Shewanellaceae* and 2 *Moraxellaceae*) expressing IMP carbapenemase without supplemental ZnSO_4_. Morganellaceae isolates had higher (*P*<0.001) MIC values than the other taxonomic family isolates.

Isolate MICs against imipenem and meropenem, with and without ZnSO_4_ added to the media, are presented in Table 1. We observed no difference in carbapenem MIC values based on supplemental ZnSO_4_ included in the media. This observation was similar for both imipenem MIC (Fig. 1) and meropenem MIC (Fig. 2). A unit of increase in the figures corresponds to a doubling of the MIC value, as graphical MIC values are log_2_ transformed. As expected, serine carbapenemase-producing control isolates were not impacted by additional zinc, and MIC values were consistent for three control isolates expressing *bla*_KPC_ with and without additional zinc for imipenem and meropenem (data not shown). *Morganellaceae* imipenem MIC without additional ZnSO_4_ present was higher (*P*<0.001) than other taxonomic families (Fig. 3), which is consistent with expected intrinsic carbapenem resistance [21].

## Discussion

We found no difference in paired carbapenem MIC values with the addition of ZnSO_4_ to the media for the inoculation of Sensititre microdilution panels. Therefore, we conclude that supplemental ZnSO_4_ does not influence the phenotypic presentation of the enzymatic activity of these MBL *bla*_IMP_ gene alleles. Our results suggest that there is adequate available zinc for effective IMP MBL hydrolysis of carbapenems in the current guidelines for MIC determination, and there is no need to recommend supplemental zinc in the media for isolates expressing *bla*_IMP_.

Available zinc in media can vary and impact the interpretation of enzymatic efficiencies [9,22,24]. It has been previously reported that variability in zinc concentration in commercial MH media can impact carbapenem MICs of *Enterobacteriaceae* that produce MBLs, indicating a need for standardization of zinc levels in media [9]. They observed that media with inadequate zinc resulted in higher meropenem MICs, with enough variability to influence the clinical interpretation [9]. We did not measure the zinc concentration of the MH broth that we used for this study, and it was not reported by the manufacturer, but our results indicate that zinc was not limiting for determining carbapenem MICs. This suggests that beyond a threshold concentration of available zinc, there is no additional benefit of supplemental zinc for the determination of carbapenem MICs in strains that produce IMP carbapenemase.

It is possible that intrinsic resistance and not carriage of *bla*_IMP_ has the more important impact on our observed differences in the MIC results. Intrinsic antimicrobial resistance is expected in some WT bacterial isolates and is therefore representative of the species. CLSI notes that *Morganellaceae*, *Proteus* spp., *Providencia* spp., and *Morganella* spp. may have intrinsic resistance, and thus elevated MICs to imipenem that make the wild-type of the species appear resistant [21]. CLSI further notes that this is likely from mechanisms other than the production of carbapenemase [21].

The overall observed difference in MIC to imipenem compared with meropenem for *Enterobacteriaceae*, *Shewanellaceae* and *Moraxellaceae* may indicate that meropenem is a stronger substrate, regardless of additionally available zinc (Table 1). The similarity of imipenem and meropenem MICs in *Morganellaceae* suggests that it is intrinsic resistance and not carriage of *bla*_IMP_ that produces the phenotypic results. Such intrinsic resistance to imipenem could be from reduced membrane permeability as has been noted in previous studies [25] or other differences unique to the *Morganellaceae* taxonomic family.

Others have reported meropenem to be a better substrate for IMP-27, as it is considered part of the IMP-6-like alleles and has a glycine aa residue substitute for serine, at position 262, compared with IMP-1 [26]. However, the studies indicating increases in MIC for meropenem compared with imipenem (among S262G alleles, i.e. IMP-6 and IMP-27) did not mention whether *Morganellaceae* were included in the analysis [26]. That IMP-27 and IMP-64 are both found in the *Morganellaceae* family in this study (and differ by only one SNP), their increase in imipenem resistance could be due to intrinsic resistance, other than the presence of the carbapenemase alone. Our sequenced *Morganellaceae* isolates did not harbour any other known carbapenemase genes, which could also contribute to reduced susceptibility to imipenem.

A study by Segawa *et al*. also examined IMP-6, where reduction in MICs to imipenem compared with meropenem was evidenced, but the IMP-6 were mostly in *Escherichia coli* and some *Klebsiella* spp. although the authors did not indicate a species-level association with MIC differences [27]. This is despite positive Carba NP tests for IMP-6 carbapenemase production, which was not accurately reflected in the MIC results for imipenem, but did correlate with the activity of the enzymatic hydrolysis [27].

Other resistance mechanisms could be influencing the observed MICs to imipenem and meropenem in *Morganellaceae*, including reduced outer membrane permeability [25]. For example, mutations resulting in reduced permeability of the ImpR outer membrane porin of *Proteus mirabilis* resulted in decreased susceptibility to carbapenems [28]. *Morganellaceae* may also exhibit intrinsic resistance to carbapenems using a combination of DHA-like beta-lactamase production and low target affinity [29]. However, these intrinsic mechanisms are likely not fully effective, which may lead these strains to sometimes acquire mobile carbapenemase genes including *bla*_IMP_. Our data are consistent with what has been reported by laboratory standard setting organizations (e.g. CLSI), indicating that *Morganellaceae* have intrinsic resistance to imipenem, independent of acquired carbapenemase-mediated resistance, which appears to be independent of supplemental zinc availability. A complete understanding of the mechanisms of intrinsic and acquired resistance in potentially pathogenic bacteria is critical to appropriate antimicrobial treatment recommendations and effective antimicrobial stewardship. Our results suggest the need for continued research in this area.

We have also potentially identified a substrate preference for the *bla*_IMP_ alleles for meropenem over imipenem among non-*Morganellaceae* isolates, supporting findings reported for *Enterobacteriaceae* by others for *bla*_IMP-6_ and *bla*_IMP-6-like_ alleles [2,26, 27].

However, the phenotypic results we observed may not be indicative of the level of bacterial *bla*_IMP_ gene expression. Meini *et al*. [26] suggested that MIC differences in substrate preferences, reported by Yano *et al*. [23] and Liu *et al*. [22], could be attributed to variations in enzymatic assays. In particular, the differences in zinc concentration in the two studies (0.01 mmol ZnCl_2_ versus 0.1 mmol ZnSO_4_, respectively) could contribute to differences in catalytic efficiencies of meropenem hydrolysis of the *bla*_IMP_ under differing zinc concentrations [22,23, 26]. In MBLs including IMP, the required availability of the metal co-factor, zinc, is critical for the enzyme to be fully active *in vivo*, particularly during the time of folding, which occurs after translocation from the cytoplasmic membrane to the periplasm [30,32]. Some experiments have shown that for MBLs to be active, they are dependent upon the concentration of extracellular zinc, as periplasmic zinc availability is not well regulated and is determined by extracellular concentrations [7]. Extracellular zinc is an important consideration in times of infection when host immune responses may restrict the availability of this important micronutrient [7]. Adding to the complexity of understanding the bacterial phenotypic expression of antimicrobial resistance and the availability of extracellular zinc, commercially available cation-adjusted MH broth varies in zinc cation concentration enough to impact the interpretation of MIC values for MBLs [9,24]. Also complicating the need for better *in vitro* testing to better approximate *in vivo* enzymatic reactions, most dilutions in cation-adjusted media may exceed physiological concentrations of zinc in patients [7]. Therefore, current phenotypic testing likely cannot adequately predict the clinical impacts of MBLs [7], especially those from taxonomic families other than *Morganellaceae* that may be intrinsically susceptible to imipenem.

Our results are directly applicable to research laboratories testing MICs of environmental isolates producing IMP carbapenemase. However, this result also suggests that supplemental zinc may be unnecessary for MIC testing of clinical isolates when cation-adjusted MH broth has adequate available zinc. The implication for clinical laboratories, including those where IMP-mediated resistance is emerging, is that standard commercial media are appropriate for determining MICs of isolates expressing *bla*_IMP_.

Our results are limited by the small sample size used for this study, but isolates expressing *bla*_IMP_ are rare and additional isolates were not available for this project. However, it is unlikely that even with a much larger sample size, we would have detected such a small observed difference in MIC values by ZnSO_4_ supplementation. In addition, we utilized only environmental and livestock commensal isolates for this study, which may not be representative of clinical diagnostic isolates. Future research utilizing clinical isolates might provide additional insight into the role of supplemental zinc in the determination of clinical diagnostic MICs. There is also still a need for rapid diagnostic testing to detect the presence of MBLs by bacterial pathogens and for bacterial speciation, as resistance to carbapenems remains a serious public health concern [1,33].

Our results indicate that the addition of supplemental ZnSO_4_ does not affect the phenotypic expression of carbapenem susceptibility among different bacterial taxa from environmental sources that carry *bla*_IMP_. This result indicates that supplemental zinc is unnecessary for MIC determination of bacterial isolates producing IMP carbapenemase. Additional research investigating other potentially important mechanisms influencing the expression of carbapenemase genes including *bla*_IMP_ and the accurate determination of clinical MICs are needed. Treatment of carbapenem-resistant infections remains critical in sepsis patients, and the availability of accurate clinical diagnostic results is important to appropriate therapeutic decision-making.

## Abbreviations

CLSI: Clinical and Laboratory Standards Institute
MBL: metallo-beta-lactamase
MH: Mueller–Hinton
